# Experimental and Numerical Study of a Rebar-Prestressed Cylinder Concrete Pipe (RPCCP) under Internal Load

**DOI:** 10.3390/ma15217771

**Published:** 2022-11-04

**Authors:** Yueyang Sun, Yiqun Huang, Yangyang Yin, Yang Wang, Shaowei Hu

**Affiliations:** 1 School of Civil Engineering, Suzhou University of Science and Technology, Suzhou 215011, China; 2 School of Civil Engineering, Fujian University of Technology, Fuzhou 350118, China; 3 School of Civil Engineering, Shaoxing University, Shaoxing 312000, China; 4 College of Water Resources and Hydropower, Wuhan University, Wuhan 430072, China; 5 School of Civil Engineering, Chongqing University, Chongqing 400045, China

**Keywords:** rebar-prestressed cylinder concrete pipe, internal load, field test, cracking load, finite element model, tension control stress

## Abstract

In order to study the load-bearing failure characteristics of a RPCCP under internal load, a field prototype test was designed, and a finite element model was established. An internal load was applied up to 2.0 MPa step by step and the force variation law of each part was obtained. During the production of the RPCCP, by wrapping prestressed steel bars around the concrete core with a cylinder, the core was subjected to an initial precompression stress. In the loading process, the protective cover cracked first, from where the concrete core gradually changed from the initial compression state to a tension state, finally cracking from the inner and outer diameter. The stresses of the cylinder and steel bars increased steadily with the internal load and did not yield. The finite element calculation results were in good agreement with the test results, and the influence characteristics of the tension control stress of the steel bar and the concrete strength on the failure of the RPCCP under internal load were discussed. The results showed that the internal load of the protective cover was independent of the tension control stress, but decreases with a decrease in concrete strength, while the load corresponding to the concrete core entering plasticity is related to the tension control stress and the concrete strength, and the relationships were basically linear.

## 1. Introduction

**Figure 1:**
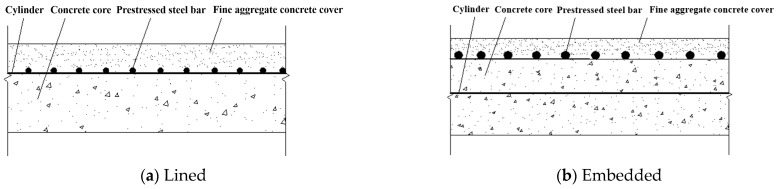
RPCCP structure.

A rebar-prestressed cylinder concrete pipe (RPCCP), which follows the Chinese standards SL702-2015 [1] and GB 50332-2002 [2] as well as American standards AWWA C303 [3] and AWWA C304 [4] for structural design and improvement, is a new pipe to meet the needs of innovative development, green low-carbon development, and high-quality development of an industry. It is a composite pipe made by using cold-rolled, ribbed, prestressed steel bars to wrap around the outside of the concrete core with a steel cylinder and creating a fine aggregate concrete protective cover. The RPCCP structure is similar to that of a prestressed concrete cylinder pipe (PCCP), which can be classified as lined or embedded according to the cylinder position. In the lined RPCCP, as shown in Figure 1a, the concrete core is poured inside the cylinder and the prestressed steel bars are wrapped around the cylinder. The inner diameter usually does not exceed 1200 mm. In the embedded RPCCP, as shown in Figure 1b, the cylinder is buried in the concrete core and the prestressed steel bars are wrapped around the outer wall of the concrete core. It is mainly used for RPCCP with an inner diameter over 1200 mm.

RPCCP has only been in use for less than 7 years and previous studies mainly focus on PCCP. In the last century, researchers such as Ross [5], Zarghamee [6,7], and Tremblay [8] took the lead in the experimental studies and revealed the loading mechanism of PCCP under an internal load. After it was introduced into China in 1989, Hu Shaowei [9] carried out prototype tests on the large-diameter PCCPs used in the South-to-North Water Diversion Project, revealing the failure rules under internal and external loads and completed the safety impact evaluation of cracks, wire breakage, and prestress relaxation in PCCP. In recent years, Dou Tiesheng [10,11], Li Yanlong [12,13], and Fang Hongyuan [14,15] have made innovations in testing methods, using FBG, PPP-BOTDA, and OFDR technologies to test the bearing performance and failure law of each component structure of a PCCP under internal or external load and when the wire is broken. Cracks in the concrete core and mortar protective cover are monitored at a spatial resolution of 10 mm. The research results can provide an effective means for long-term structure health monitoring of a PCCP by fiber-sensing technology. Prestressed steel wire is the guarantee of the strength of the whole PCCP. Once the wire is broken, the PCCP has the risk of bursting. Therefore, researchers have proposed a series of repair and reinforcement methods for a PCCP with broken wires, including the post-tensioned prestressing method, internal steel-lining method, lining and outsourcing FRP repair technology, etc. Among them, there are many studies on CFRP reinforcement, including sticking CFRP [16,17,18] or prestressed CFRP [19,20] on the inner wall of the concrete core and at the position of the broken wire. The results show that the repair of PCCP by CFRP has a remarkable effect.

Compared with PCCP, RPCCP has the following two characteristics [21,22]: (1) the thicker cold-rolled, ribbed, prestressed steel bars, with a low stress grade and yield strength of 650 MPa or 970 MPa, are used in a RPCCP; and (2) a C50 fine aggregate concrete protective cover is poured on the ribbed prestressed steel bars. If the calculation formulas for the relevant specifications of the PCCP are directly used for the design of a RPCCP, the results will have a large deviation from the actual test results. In addition, a RPCCP is a kind of pipe that has been used for less than 7 years. Few tests have placed measuring points on the cylinder and prestressed steel bars in advance during the process of production to reveal the failure laws under an internal load. Therefore, this paper used field prototype tests and a finite element model to study the bearing capacity of an embedded RPCCP with an inner diameter of 1400 mm under internal load and the circumferential strain gauges were arranged on the cylinder, steel bars, and concrete protective cover, in order to reveal the force response of each part. The research results can provide a reference for the formulation of relevant standards and specifications of a RPCCP for pipe manufacturers, and also provide a basis for the further popularization and application of RPCCPs in water transmission and diversion projects.

## 2. Experiment Design

### 2.1. Geometric Sizes and Material Parameters

The geometrical sizes and material mechanical parameters of the tested RPCCP are shown in Table 1 and Table 2, respectively. The core and protective cover were poured with C50 concrete, and the design value of the tensile strength was calculated by using Equation (1) [4]. The elastic modulus of the concrete, cylinder, and steel bar had the conventional values [23,24].

(1) $$f_{t}{}^{\prime }=0.52\sqrt{f_{\mathrm{cu},k}}$$

where *f*_cu,k_ is the standard value of the compressive strength of concrete, and *f*_t_^′^ is the design value for the tensile strength of concrete.

### 2.2. Arrangement of Testing Points

During the test, the pipe was filled with water, and the internal water pressure was evenly applied to the inner wall of the concrete core, which mainly led to longitudinal cracks along the pipe length, while circumferential cracks did not easily appear. Therefore, this study mainly arranged the testing points in the circumferential direction of the pipe body.

**Figure 2:**
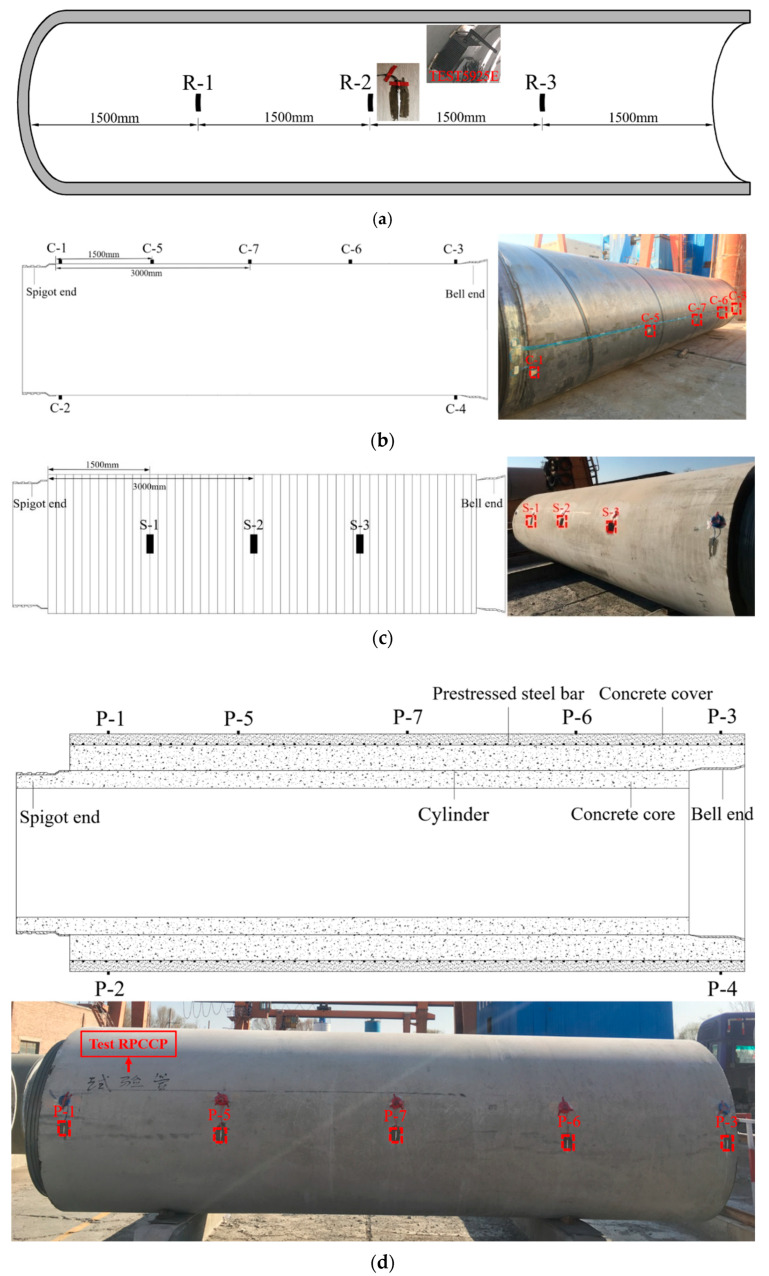
Layout of the strain gauges. (**a**) Inner surface of the core. (**b**) Cylinder. (**c**) Steel bar. (**d**) Concrete cover.

First, in the production process of the test RPCCP, three circumferential resistance strain gauges, R-1, R-2, and R-3, as shown in Figure 2a, were arranged on the inner surface of the core before the bars were wrapped. The wireless dynamic signal testing and analysis system TST5925E was also pasted on the surface, and the data of the three circumferential resistance strain gauges were collected at a frequency of 50 Hz when the wrapping was carried out.

Second, seven circumferential resistance strain gauges were arranged on the outer wall of the cylinder in advance during the production of the RPCCP. Among them, two were arranged 100 mm near the ends of the spigot and the bell, respectively, and their numbers were C-1, C-2, C-3, and C-4. Then, three sections were set at 1500 mm and 3000 mm close to the pipe ends, and a resistance strain gauge was arranged on each section, namely, C-5, C-6, and C-7. The specific arrangement numbers of the measuring points are shown in Figure 2b.

Third, in order to obtain the strain of the prestressed steel bars in the bearing-load process accurately, after the concrete protective cover was poured, three measuring points were selected to cut the concrete cover outside, and the resistance strain gauges were arranged on the prestressed steel bars, numbered as S-1, S-2, and S-3, respectively. The specific arrangement numbers of the measuring points are shown in Figure 2c [22].

Finally, a total of five sections were set at both ends of the spigot and bell, as well as 1500 mm near the ends and 3000 mm in the middle of the pipe. Seven circumferential strain gauges, numbered P-1, P-2,…, P-7, as shown in Figure 2d, were arranged to measure the stress of the concrete cover in the bearing-load process.

### 2.3. Test Device and Loading Method

**Figure 3:**
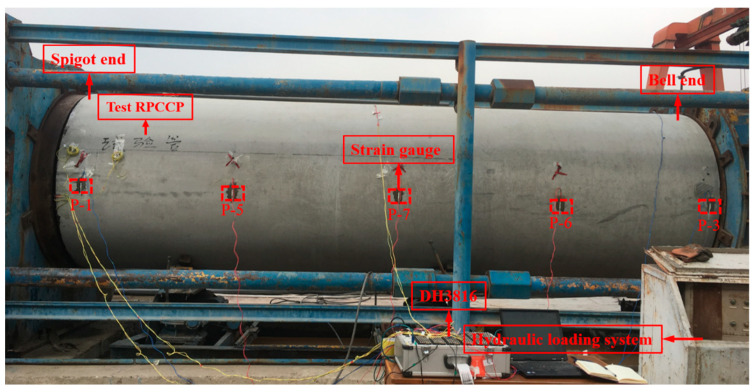
Horizontal internal hydraulic pressure test device.

The test was conducted at the production site of Ningxia Qinglong Pipe Industry Group Co., Ltd in Wuzhong, Ningxia Hui Autonomous Region, China. Since the ends of the RPCCP and PCCP have the same type of spigot and bell, the same equipment can be used when pressurized. Regarding the load, only the internal load was considered, and a horizontal pressure device was adopted, as shown in Figure 3. When installing the pipe, both ends were sealed with rubber sealing rings to prevent leakage, and water was injected into the pipe with a pressurized pump. By multi-stage loading, for every 0.05 MPa increase in load, strain values were collected after 5 min of stabilization and the appearance and development of cracks in the concrete protective cover were observed by professionals.

## 3. Establishment of Finite Element Model

### 3.1. Modeling

The RPCCP structure is similar to that of a PCCP. According to the mechanical characteristics of RPCCP and the related references on PCCP finite element models [15,25], on the basis of satisfying the analysis and improving the computational efficiency, the following simplified assumptions were made at first when establishing the model:

(1) Material nonlinearities were considered, and geometric nonlinearities were not.
(2) Suppose the deformations were small.
(3) The effects of shrinkage and creep of the concrete were not considered.

The RPCCP 3D finite element model included all the actual components of the structure. The cylinder, concrete core, prestressed steel bar, and concrete cover were modeled separately. The type of RPCCP was embedded, in which the cylinder divided the concrete core into an inner concrete core and outer concrete core. Therefore, the model consisted of an inner concrete core, a cylinder, an outer concrete core, prestressed steel bars, and a concrete protective cover from inside to outside—a structure with a total of five layers. It was very important for the reliability and correctness of the model to consider the connection between each layer reasonably. Under the action of the internal load, the failure of each layer was mainly controlled by the circumferential tensile stress, and all of them were pressurized in the radial direction. This paper dealt with the relationship between each layer according to the following measures:

(1) It was assumed that there was complete contact between the cylinder and the inner and outer concrete core, and the deformation coordination was satisfied without considering the slip and separation between layers.
(2) Due to the effect of prestress, the outer concrete core had a good bond with the prestressed steel bars, and the slip and detachment between them were not considered. In this regard, the constraint equation was used in the finite element model to coordinate the degrees of freedom of the steel bar element and the concrete element to simulate the bond.
(3) There was no treatment between the prestressed steel bars and the concrete protective cover; that is, it was assumed that when the protective cover cracked or denudated, it would not have a direct impact on the steel bars, and the bond between them was transmitted through the outer concrete core indirectly.
(4) “Tie” was used to simulate the connection between the concrete protective cover and the outer concrete core.

**Figure 4:**
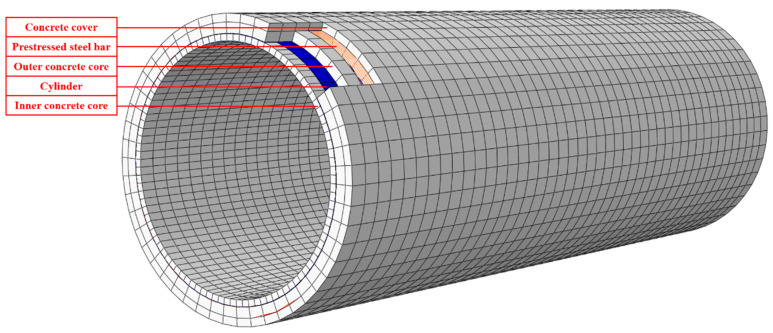
Finite element model of the RPCCP under internal load.

The concrete was simulated by solid element C3D8R, which had eight nodes, and each node had 3 degrees of freedom. The steel cylinder belonged to a thin-walled structure and was simulated by the thin shell element S4R. The prestressed steel bar was simulated using a beam element. The final finite element model is shown in Figure 4.

### 3.2. Constitutive Relationship of Materials

(1) Constitutive model of concrete

**Figure 5:**
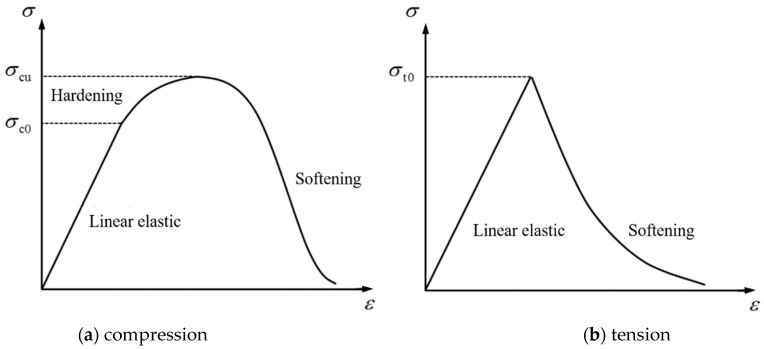
Concrete damaged plasticity model.

The concrete damaged plasticity model was adopted for the constitutive model of concrete [23]. This model took into account the differences in the tension and compression properties of the materials, which were consistent with the actual failure modes, including compression crushing and tension cracking. The nonlinear behavior of the concrete was simulated by combining isotropic elastic damage and plastic tension and compression. The stress–strain curve of compressive damage is shown in Figure 5a. The initial stage is the linear elastic stage. When the compressive yield strength is reached, the relationship turns into a curve and enters the hardening stage until the ultimate compressive strength is reached, followed by the softening stage. The initial tensile stage is the same as the initial compression stage, which is the linear elastic stage, as shown in Figure 5b. After reaching the max stress, it enters the softening stage. The elastic moduli of compression and tension are the same, while the damage factors are different.

The stress–strain relationship of the concrete after compression damage is shown in Equation (2), where *σ*_c_ is the compressive stress of concrete, *d*_c_ is the compressive damage coefficient, the value range is 0~1, *d*_c_ = 0 indicates that the concrete has no compressive damage, *d*_c_ = 1 indicates complete compression failure, *E*_c_ is the elastic modulus of the concrete, *ε*_c_ is the compressive strain of the concrete, and $\varepsilon_{c}^{\mathrm{pl}}$ is the compressive plastic strain of the concrete.

(2) $$\sigma_{c}=\left(1-d_{c}\right)E_{c}\left(\varepsilon_{c}-\varepsilon_{c}^{\mathrm{pl}}\right)$$

The stress–strain relationship of the concrete after tension damage is shown in Equation (3), where *σ*_t_ is the tensile stress of the concrete, *d*_t_ is the tensile damage coefficient, the value range is 0~1, *d*_t_ = 0 indicates that the concrete has no tensile damage, *d*_t_ = 1 indicates complete tension failure, *ε*_t_ is the tensile strain of the concrete, and $\varepsilon_{t}^{\mathrm{pl}}$ is the tensile plastic strain of the concrete.

(3) $$\sigma_{t}=\left(1-d_{t}\right)E_{c}\left(\varepsilon_{t}-\varepsilon_{t}^{\mathrm{pl}}\right)$$

(2) Constitutive model of prestressed steel bar

**Figure 6:**
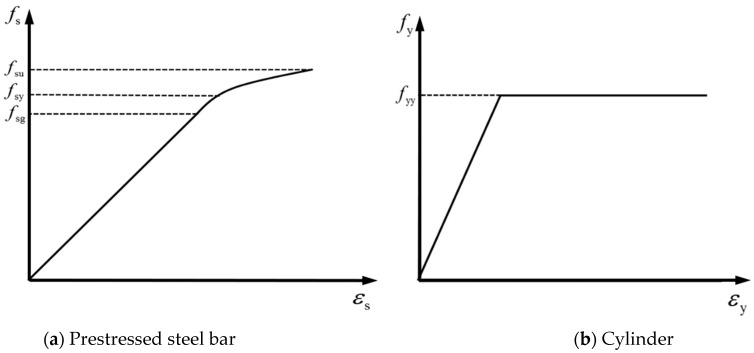
Stress–strain curves of the prestressed steel bar and cylinder.

The constitutive model of the prestressed steel bar adopted the following stress–strain relationship [4], as shown in Figure 6a:

(4) $$\begin{array}{l} f_{s}=\varepsilon_{s}E_{s}\\ f_{s}=f_{\mathrm{su}}\left\{1-{\left[1-0.6133\left(\varepsilon_{s}E_{s}/f_{\mathrm{su}}\right)\right]}^{2.25}\right\} \end{array}\begin{array}{l} \;\;\;\;\;\varepsilon_{s}\leq f_{\mathrm{sg}}/E_{s}\\ \;\;\;\;\;\varepsilon_{s}>f_{\mathrm{sg}}/E_{s} \end{array}$$

we *f*_su_ is the standard value of the tensile strength, *f*_sg_ is the control stress for prestressing, and the yield stress of the prestressed steel bar is 85% of its tensile strength, *f*_sy_ = 0.85 *f*_su_.

(3) Constitutive model of cylinder

According to the data provided by the manufacturer, the design value of the tensile yield strength of the cylinder was *f*_yy_ = 235 MPa, and the ideal elastic-plastic stress–strain relationship was adopted, as shown in Figure 6b [13].

### 3.3. Simulation of Prestress

In finite element models, there are usually four methods to simulate the prestress of steel bars: equivalent load method, initial strain method, initial stress method, and equivalent cooling method [14]. The equivalent cooling method was selected to simulate the prestress of steel bars in the RPCCP. This method uses the principle of equivalent deformation to reduce a certain temperature and prestress the steel bar through the thermal expansion and contraction, which considers the contribution of the stiffness of the steel bars to the whole structure, and adjusts the cooling value according to the losses. The calculation formula is as follows:

(5) $$\Delta t=\frac{f_{\mathrm{sg}}}{\alpha_{t}E_{s}}$$

where Δ*t* is the amount of cooling that needs to be applied, *f*_sg_ is the control stress for prestressing, *E*_s_ is the elastic modulus of the prestressed steel bar, and *α*_t_ is the linear expansion coefficient, which is taken as 0.0001 in this paper.

### 3.4. Analysis of Mesh Sensitivity

**Figure 7:**
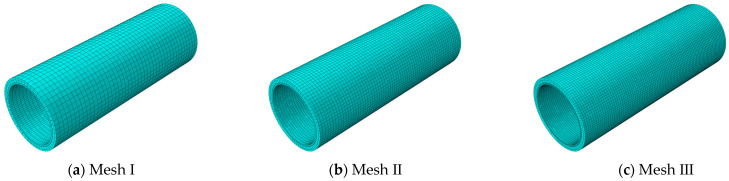
Three different mesh configurations with a different mesh density.

In order to make the results of the finite element model more reliable and strike a balance between accuracy and computational efficiency, an appropriate mesh density should be selected. Take the concrete model in the protective cover and the outer concrete core as an example, the circular cross section was divided into different number of meshes—50, 75, and 100—and the corresponding concrete model in the inner concrete core was divided into 100, 150, and 200 in the circular cross section. The models were named Mesh Ⅰ, Mesh Ⅱ, and Mesh Ⅲ from left to right, as shown in Figure 7. Mesh Ⅰ had 19,900 elements and 27,501 nodes, and the numbers of Mesh Ⅱ and Mesh Ⅲ were 31,782 and 48,627 and 68,100 and 97,851, respectively.

**Figure 8:**
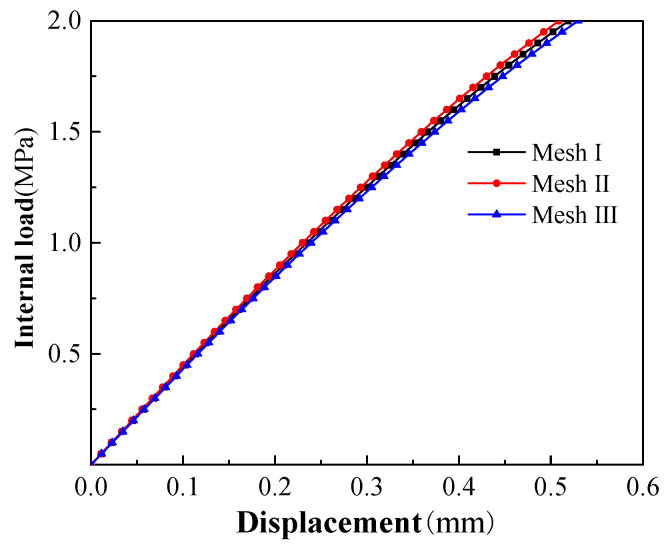
Internal load–displacement curve of the concrete cover.

In the three models, a node of the concrete unit of the protective cover in the middle of the pipe body was taken as an example, and the variation curves of displacements with the internal load are shown in Figure 8. It can be seen that the three internal load–displacement curves obtained under different mesh densities basically coincided, and the deviation of the displacements after the concrete entered the plastic stage were slightly larger than those in the elastic stage. Based on the comprehensive consideration of the calculation time and the accuracy of the results, the configuration of Mesh Ⅰ was selected for subsequent analysis and calculation.

## 4. Results and Discussion

### 4.1. Testing Phenomenon

In the initial stage of loading, the pressure was small, the force of the whole pipe was uniform, and no visible cracks appeared in the concrete cover. When the internal pressure was loaded to 1.5 MPa, the first visible small crack, with a length of 140 mm, appeared in the protective cover near the end of the spigot, indicating that the concrete cover began to crack. The internal load 1.5 MPa was the initial cracking load.

**Figure 9:**
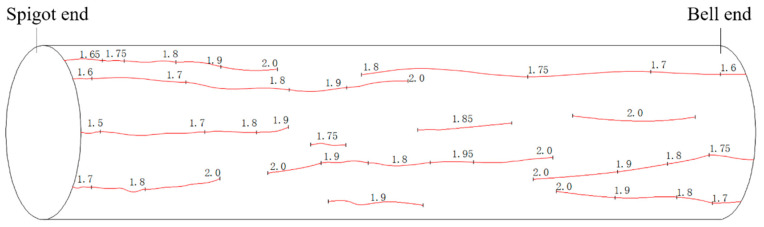
Distribution and expansion of cracks in the concrete protective cover over half of the pipe body.

As the load increased, the second and third cracks appeared at 1.6 MPa, and the first crack expanded, with its width increasing. When the internal load reached 1.75 MPa, several cracks appeared in the pipe body, which was finally pressurized to 2.0 MPa due to the capacity of the pressurizing device and safety considerations. The bearing capacity of the RPCCP under the internal load was significantly higher than 2.0 MPa. The distribution and expansion diagram of the cracks in the protective cover of half of the pipe body is shown in Figure 9. The numbers beside the cracks represent the values of the load at the time when the cracks appeared or expanded.

### 4.2. Whole-Process Strain Analysis

(1) Wrapping process

**Figure 10:**
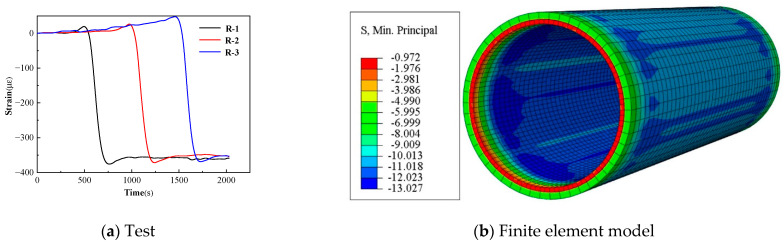
Strain of the concrete core after wrapping with steel bars.

The results of the field wrapping test are shown in Figure 10a, with positive tension and negative compression. The abscissa of the image is the time of wrapping, and the ordinate is the circumferential compressive strain on the inner surface of the concrete core. According to the curve, the steel bars wrapped the concrete core passing through measuring point R-1 first, and the strains of the three measuring points basically showed the same trend. The final circumferential compressive strain was about 350 με, which was 11.34 MPa after conversion. In the specification [1], it is assumed that the circumferential stress at each part of the pipe section is distributed uniformly. The equilibrium equation is established through the tension of the steel bars and the compression of the concrete core and cylinder, so the stress of each part after wrapping can be obtained according to the deformation coordination. After calculation, the initial compressive stress of the concrete core was 12.18 MPa. The results of the finite element model are shown in Figure 10b and the numerical unit is MPa. The maximum compressive stress on the inner surface of the concrete core was 13.03 MPa. The loss of prestress was not taken into account when using the specification method and the finite element model. It can be seen that the field test result was the smallest, because it was difficult to ensure the absolute circumferential direction of the strain gauges pasted on site; a slight deviation would lead to a smaller final measurement result. The result of the specification was smaller than that of the finite element model, because the specification assumed that the circumferential precompression stress of the concrete core was equal everywhere along the thickness direction, and the calculation result was similar to the average value of the circumferential stress that can reflect the inner and outer surface of the concrete core. In general, the results obtained by the three methods were close to each other, and it is feasible to use any of these methods to calculate the precompression stress of the concrete core.

(2) Strain variation law of concrete cover

**Figure 11:**
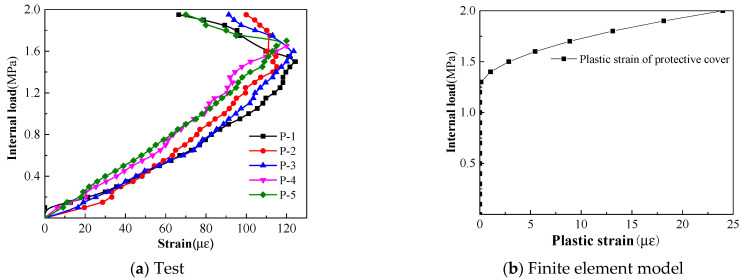
Internal load–strain curve of the concrete cover.

The strain curves of the concrete cover under internal load are shown in Figure 11a. Due to the roughness of the protective cover, uneven coating of the epoxy resin, and the fact that the test was carried in an open field, the external factors had a significant influence. Five measuring points, namely, P-1, P-2, P-3, P-4, and P-5, with good change regularity, were selected for analysis. It can be seen from the curves that the strains increased steadily before the internal load was 1.4 MPa. When the load reached 1.45 MPa, the measuring point P-2 reached the maximum tensile strain. As the load continued to increase, the collection status of P-4 in the subsequent collection showed “overflow”, because the crack just appeared at the position where the strain gauge was pasted, and the strain gauge was damaged. The strains at P-1, P-2, P-3, and P-5 decreased, because the cracking occurred near the measuring points and the stresses were released. Compared with the other measuring points near the spigot or the bell end, the concrete cover at P-5 cracked later and reached the maximum tensile strain when the load was 1.7 MPa. According to Table 2, the strain corresponding to the design value of the tensile strength of the concrete is 113 με, and the maximum tensile strain measured in this test is 124 με, which is in good agreement with each other. The step loading and static strain collection system were adopted, which was different from the dynamic strain collection system. The maximum tensile strain during the loading process may be generated in the process of applying a certain level of internal load, which was difficult to be measured by the static strain collection system.

The visible crack of the concrete cover was taken as the failure criteria in the test, and entering plasticity was taken as the failure criteria in the finite element model. According to the tensile constitutive model of concrete in Figure 5b, entering plasticity means that concrete enters the softening stage after tensile yield. For the C50 concrete constitutive model used in this paper, the tensile stress reached 2.64 MPa and then entered the softening stage; that is, it enters the plastic stage. Just entering plasticity does not mean that the concrete begins to crack. Cracking is usually generated after plastic accumulation to a certain process, so the result of the finite element model is smaller than those of the test. A node in a unit of the concrete cover model is taken for analysis, and the curve of the plastic strain with internal load is shown in Figure 11b. It can be seen that the plastic strain began to appear when the load reached 1.4 MPa; that is, the protective cover began to enter plasticity, which was smaller than the cracking load of 1.5 MPa in the test.

According to the theory of uniform pressure inside the ring or cylinder in elasticity, the tensile stress on the inner surface should be the largest, and the inner surface of the concrete core should be the first to crack under tension. However, in the process of bearing internal load, the concrete cover cracked before the concrete core. During manufacturing, the concrete core was wrapped by the prestressed steel bars so that it was subjected to an initial precompression stress, and then the outermost concrete cover was poured but which was not subject to any force. Therefore, when the RPCCP began to bear an internal load, the concrete protective cover was tensioned directly, and the initial compressive stress of the concrete core would offset some of the internal load.

(3) Strain variation law of cylinder

**Figure 12:**
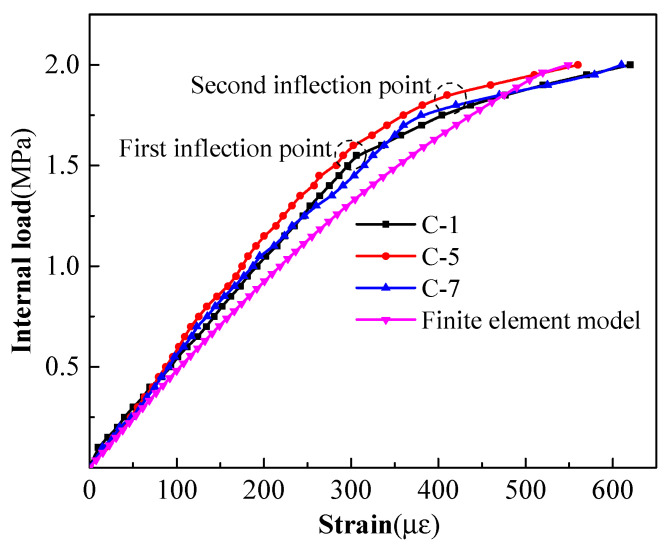
Internal load–strain curve of the cylinder.

After the cylinder was manufactured, seven strain gauges were pasted on the surface. However, because it was buried in the concrete core, which should be vibrated during pouring, some strain gauges failed. In addition, the cylinder and the concrete core rotated together during wrapping, and the strains were not measured until the internal load was applied. Finally, effective strain data were collected at measurement points C-1, C-5, and C-7. Moreover, the cylinder was initially subjected to the precompression stress caused by the prestressed steel bars, so the measured strains in the test were not the true strains. Meanwhile, in order to facilitate comparison, the results of a node in a unit of the cylinder model after wrapping were zeroed and the internal load–strain relationship curves of the test and the finite element model are shown in Figure 12. It can be seen from the curve that the variation trends of the field test results were basically similar to that of the finite element model and the test results presented an obvious three-stage variation trend with an increase in internal load. The strain measured at about 1.5 MPa was the inflection point of the first and second stages, and the strain measured at about 1.8 MPa was the inflection point of the second and third stages. The strain at each stage increased linearly with the increase in internal load, but the increase in the third stage was obviously larger than those in the first two stages, with an increase of 0.1 MPa. It can be known from the above that the internal load corresponding to the cracking of the concrete cover was 1.5 MPa, so it can be inferred that the second inflection point of 1.8 MPa corresponded to the load before the cracking of the concrete core; that is, when the internal load was 1.9 MPa, the concrete core cracked.

The prestress loss would occur inevitably after wrapping prestressed steel bars. The calculation of loss referred to the specification [26], which mainly considered the circumferential prestress loss caused by the stress relaxation of the steel bars, shrinkage, creep, and elastic compression of the concrete. After calculation, the total prestress loss of the test RPCCP was 131 MPa, so the effective tensile stress of the prestressed steel bars was 337 MPa, and the initial compressive stresses of the concrete core and cylinder were 9.51 MPa and 57.87 MPa, respectively. According to the relevant material parameters in Table 2, the initial compressive strain of the cylinder was 276 με. The maximum tensile strain measured under the internal load of 2.0 MPa was 620 με, which was 344 με after subtracting, far less than the yield strain corresponding to the tensile yield strength, indicating that the cylinder did not yield under the internal load of 2.0 MPa. It can also be verified from the curve that the strain in the third stage still showed a linear relationship with the increase in internal load.

(4) Strain variation law of steel bars

**Figure 13:**
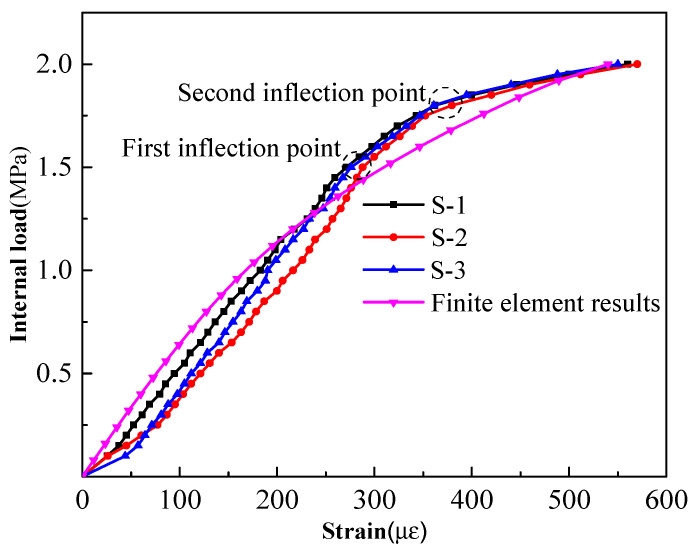
Internal load–strain curve of the prestressed steel bars.

A node in a unit of the steel bar model was selected, and the calculation results were also zeroed. The internal load–strain curves of the test and finite element model are shown in Figure 13. It can be seen from the curve that the strain data of the three measured points in the test were basically similar to those of the finite element model, and the variation trends were basically the same. The strains showed an obvious three-stage variation trend with the increase in internal load, and the loads corresponding to the inflection points were also 1.5 MPa and 1.8 MPa, respectively.

Similarly, the strains measured during the test were not the true strains of the steel bars, because they had an initial effective tensile stress of 337 MPa and the corresponding initial tensile strain was 1664 με. Under the internal load of 2.0 MPa, the maximum tensile strain measured was 570 με, and the total tensile strain after superposition was 2214 με, which was far less than the yield strain of 3137 με corresponding to the yield strength of 650 MPa. There was no yield failure in the steel bars and the whole pipe could withstand a greater internal load.

### 4.3. Analysis of Related Parameters

It can be seen from Section 4.2 that the calculated results of the finite element model are in good agreement with the test results, indicating that the model is reasonable. Therefore, on the basis of the model, this section keeps the pipe size, the grade, and the spacing of the steel bars unchanged, and only changes the tension control stress and concrete strength to analyze the influence of these two factors on the bearing performance of the RPCCP.

(1) Effect of tension control stress

**Figure 14:**
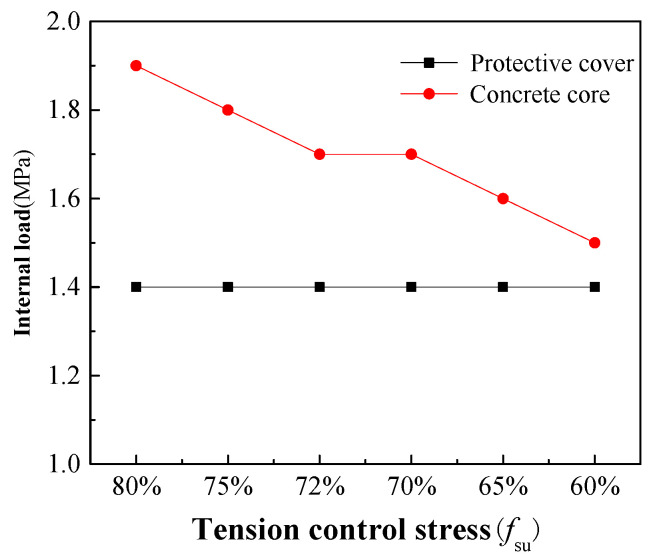
Internal loads corresponding to protective cover and concrete core when entering plasticity under different tension control stresses.

It can be seen from Table 2 that the tensile control stress of the steel bars used in RPCCP in the prototype test and model is 72% *f*_su_, where *f*_su_ is the standard value of the tensile strength of the bars. In order to study the influence of tension control stress on the bearing performance of the RPCCP, five different tension control stresses (60% *f*_su_, 65% *f*_su_, 70% *f*_su_, 75% *f*_su_, and 80% *f*_su_) were added, and the internal load was applied until the concrete core entered plasticity. The calculation results are shown in Figure 14. It can be seen that the tension control stresses have no effect on the internal loads when the concrete protective cover enters plasticity, which are in line with the reality. Although the tension control stresses are different, the pipe size and the material parameters (elastic modulus, Poisson’s ratio, etc.) and usage amount of each layer are the same; that is, the pipe stiffnesses calculated according to the size and material parameters are the same. The protective cover is cast after wrapping the prestressed steel bars and is not subjected to any force at first. Once the internal load is applied, the protective cover is tensioned in the annular direction directly. In the elastic stage before any damage to the whole pipe, the deformation increases linearly with the increase in the internal load, which has nothing to do with the tension control stress of the steel bars. Therefore, the internal loads corresponding to the cracking of the protective cover are the same, which are all 1.4 MPa in Figure 14. However, the internal loads corresponding to the concrete core entering plasticity are related to the tension control stress, and the relationship is basically linear, because the greater the tension control stress, the greater the initial compressive stress in the core, and the greater the internal load that can be offset.

(2) Effect of concrete strength

**Figure 15:**
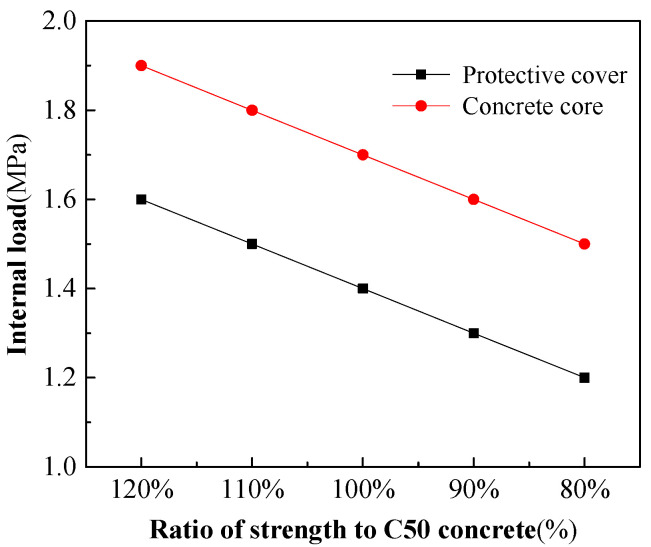
Internal loads corresponding to the protective cover and concrete core when entering plasticity under different concrete strengths.

The concrete used in the prototype test and model has a strength grade of C50. The strength, including compressive and tensile properties, certainly has an impact on the bearing capacity of the RPCCP. On the basis of the strength of the C50 concrete and keeping other conditions unchanged, the compressive and tensile properties were multiplied by four coefficients (120%, 110%, 90%, and 80%) to carry out the model calculation, respectively. The results are shown in Figure 15. The internal loads when the protective cover and concrete core enter plasticity are basically linear with the concrete strength, and the load decreases by 0.1 MPa for every 10% reduction in strength. Under an internal load, the concrete mainly appears to have tensile failure. The higher the tensile strength, the greater the load required to enter plasticity.

In this section, through the calculation of finite element models, the influence laws of the tension control stress of the steel bar and concrete strength on the protective cover and concrete core are obtained; the influence of these two factors on the yield of cylinder and steel bars were not analyzed. When they yield, the corresponding internal loads are very high, and by then the concrete has long failed and is out of service. In the actual test, the two layers were not loaded to yield, and the strength of the concrete has little influence on the internal loads corresponding to the yield of the cylinder and steel bars. The smaller the initial tension control stress is, the larger the difference between its value and the yield strength is. Because the yield strength of the steel bar is fixed, a greater internal load is required for the steel bar to yield.

### 4.4. Summary and Analysis

It is stipulated in the specification [27] that the anti-cracking internal load test of PCCPs should be carried out under the condition of standard combination of cracking control. The inspection internal load is calculated by Equation (6). It is required that under this load, the pipe body shall not burst, nor appear locally convex or show other leakage phenomena, and the cement mortar protective cover shall not have any cracks or other peeling phenomena.

(6) $$P_{\mathrm{ti}}=\frac{A_{s}f_{\mathrm{sr}}+\alpha f_{\mathrm{tk}}A_{n}}{br_{0}}$$

where *P*_ti_ is the calculated anti-cracking internal load of the test; *A*_s_ is the area of circumferential prestressed steel bars in unit length; *f*_sr_ is the final effective prestress of the circumferential steel bars; *A*_n_ is the converted area of the concrete core, cylinder, steel bars, and mortar protective cover in unit length; *α* is the controlling cracking coefficient of the mortar, which is 1.06 for an embedded PCCP and 0.65 for a lined PCCP; *f*_tk_ is the standard value of the tensile strength of the concrete; *b* is the calculated axial length of the pipe, with the value being 1000 mm; and *r*_0_ is the calculated radius of the pipe wall section.

After calculation and summary, the calculated anti-cracking internal load of the test, the cracking loads of the protective cover, the cracking loads of the concrete core, and the yield loads of the cylinder and the prestressed steel bars are shown in Table 3.

The designed working pressure of the test RPCCP is 1.0 MPa, while the calculated anti-cracking internal load of test is 1.42 MPa, which is larger than the design working pressure. The cracking loads of the concrete cover obtained by the test and the finite element model are also 0.4~0.5 MPa larger than the design working pressure, indicating that each layer of the RPCCP under the design working pressure is in the elastic stage. The larger the difference between the test and finite element results and the design working pressure, the less likely the protective cover is to crack and the larger the safety stock is in the actual operation.

The results of the finite element model are smaller than those of the actual test, because entering plasticity is taken as the failure criteria in the finite element model, which does not mean that the concrete begins to crack. Cracking is usually generated after plastic accumulation to a certain process. The cracking load of the concrete core in the test RPCCP is 0.4 MPa higher than that of the protective cover. In the finite element model of the RPCCP, the internal loads corresponding to the yield of the cylinder and the steel bar are very close, indicating that the material selection and size design of the RPCCP are reasonable.

## 5. Conclusions

In this paper, the initial cracking load of the RPCCP with an inner diameter of 1400 mm under internal load was obtained by the field test and finite element simulation. Several conclusions can be obtained as follows:

(1) In the process of RPCCP production, strain gauges are pre-arranged to obtain the cracking loads of the protective cover and concrete core and the force variation characteristics of the cylinder and prestressed steel bars. Finally, the failure law of the RPCCP under internal load is revealed. During the loading process, the concrete protective cover cracks first, form where the concrete core gradually changes from the initial compression state to the tension state, and finally cracks from the inner and outer diameter. The cracking load is greater than that of the protective cover.
(2) The finite element model of RPCCP under internal load is established by selecting the appropriate material constitutive relationships and a reasonable mesh density and simulating the prestress by the equivalent cooling method. The whole process of RPCCP from the application of prestress to the final failure was analyzed. The finite element results were compared with the test results to verify the rationality of the model, and the influence characteristics of the tension control stress of the steel bars and the concrete strength on the failure of the RPCCP under internal load were discussed.
(3) The calculated anti-cracking internal load of the RPCCP according to the specifications is consistent with the results of the test and finite element model, which are all higher than the design working pressure, indicating that each layer of the RPCCP under the design working pressure is in the elastic stage. The larger the difference between the test and finite element results and the design working pressure, the less likely the protective cover is to crack and the larger the safety stock is in the actual operation.

In this study, the failure law of the RPCCP under internal load is revealed through a field prototype test and finite element model. However, the RPCCP in actual operation must bear an external load as well as internal load, so it is necessary to carry out research on the mechanical performance of the RPCCP under external load. In addition, this study made some assumptions and simplifications when establishing the finite element model of the RPCCP. The reliability of this model in calculating the bearing performance of the RPCCP under external load needs to be further studied.

## Figures and Tables

**Table 1:** Geometrical sizes of the RPCCP.

Type	Designed Working Pressure/MPa	Inner Diameter/mm	Thickness of Concrete Core/mm	Thickness of Cylinder/mm	Inner Diameter of Cylinder/mm	Thickness of Concrete Cover/mm	Bar Diameter/mm	Spacing of Bars/mm
Embedded	1.0	1400	110	2	1504	60	8	18

**Table 2:** Mechanical parameters of the materials used in the RPCCP.

Elastic Modulus of Concrete *E*_c_/(N/mm^2^)	Design Value of Tensile Strength of Concrete *f*_t_^’^/MPa	Standard Value of Tensile Strength of Bars *f*_su_/MPa	Tension Control Stress	Elastic Modulus of Bars *E*_s_/(N/mm^2^)	Tensile Yield Strength of Cylinder *f*_yy_/MPa	Elastic Modulus of Cylinder *E*_y_/(N/mm^2^)
32,500	3.68	650	72%*f*_su_	205,000	235	210,000

**Table 3:** Results of bearing an internal load.

Pipe	Inner Diameter/mm	Research Method	Calculated Anti-Cracking Internal Load of Test/MPa	Cracking Load of Protective Cover/MPa	Cracking Load of Concrete Core/MPa	Yield Load of Cylinder/MPa	Yield Load of Prestressed Steel Bars/MPa
RPCCP	1400	Test	1.42	1.5	1.9	Not measured	Not measured
		Finite element model		1.4	1.7	3.0	3.1
